# Quantitative approach to realization of ultrasonic grain refinement of Al-7Si-2Cu-1Mg alloy

**DOI:** 10.1038/s41598-019-54161-7

**Published:** 2019-11-28

**Authors:** Soo-Bae Kim, Young-Hee Cho, Min-Su Jo, Jae-Gil Jung, Young-Kook Lee, Jung-Moo Lee

**Affiliations:** 1 Metallic Materials Department, Korean Institute of Material Science, Changwon, 51508 Republic of Korea; 2 Department of Materials Science and Engineering, Yonsei University, Seoul, 120-749 Republic of Korea

**Keywords:** Engineering, Materials science

## Abstract

Ultrasonic melt treatment (UST) was applied to Al-7Si-2Cu-1Mg melt at various temperatures of 620, 650, 700 and 785 °C. MgAl_2_O_4_ particles which were often found to be densely populated along oxide films, became effectively dispersed and well-wetted by UST. Transmission electron microscopy work combined with crystallography analysis clearly indicates that MgAl_2_O_4_ particles can act as α-Al nucleation site with the aid of UST. However, with UST, grain refinement occurred only at temperature of 620 °C and the grain size increased from 97 to 351 μm with increase of melt temperature to 785 °C for UST. In quantitative analysis of grain size and MgAl_2_O_4_ particle diameter, it was found that ultrasonic de-agglomeration decreased mean particle size of the MgAl_2_O_4_ particles, significantly reducing size from 1.2 to 0.4 μm when temperature increased from 620 to 785 °C. Such a size reduction with increased number of MgAl_2_O_4_ particles does not always guarantee grain refinement. Thus, in this work, detailed condition for achieving grain refinement by UST is discussed based on quantitative measurement. Furthermore, we tried to suggest the most valid grain refinement mechanism among the known mechanisms by investigation of the relationship between grain size and particle size with variation of melt temperature.

## Introduction

The grain refinement of aluminum and its alloys has long been approached since it involves such benefits as reduced segregation, enhanced feeding of liquid in the mushy zone, and reduced or better-dispersed porosity; thus has been often achieved through the addition of nucleant particles^1^. Besides inoculation, various liquid metal processes including mechanical shearing^2^, ultrasonic melt treatment^3–5^ and subsequent rapid solidification have been suggested to enhance the heterogeneous nucleation by evenly distributing the nuclei particles as well as triggering nucleation of α-Al grains with substantial undercooling.

Among these, for aluminium alloy casting, inoculation using Al-Ti-B system master alloys is the most widely used method to achieve grain refinement^6,7^. TiB_2_ particles present in Al-Ti-B alloys act as nucleation site for α-Al grains, promoting heterogeneous nucleation. The grain refinement performance by inoculation depends on the potency, number and size of nucleant particles, and on undercooling. The number of potent particles can be determined by the particle size. Quested *et al*.^7^ and Greer *et al*.^6^ estimated the relationship between TiB_2_ particle size and α-Al grain size when total volume of TiB_2_ in the melt is constant. In their reports, the number of particles increases as the mean particle diameter decreases and this leads to a decrease in the grain size. According to the free growth theory, required undercooling for nucleation is inversely proportional to the particle size and can be estimated using the following equation

1 $${d}^{\ast }\ge d=\frac{4\gamma }{\Delta {S}_{v}\Delta {T}_{g}}$$

where *γ* is the solid-liquid interfacial energy, Δ*S*_*v*_ is the entropy of fusion per unit volume and *d* and d* are diameter and critical diameter of particles respectively^6,8^. At the given undercooling, Δ*T*_*g*_, only particles larger than the critical diameter (*d* ≥ d*) can be activated for nucleation. Equation 1 also implies that an increase in undercooling can also increase the number of potent particles. Recently, Fraś *et al*.^9^, based on a quantitative measurement of grain size and the particle size, showed that the number of activated particles for α-Al nucleation increases as the degree of undercooling increases. In addition, from the relationship between the number of activated particles and the undercooling, they estimated the critical diameter of TiB_2_ for α-Al nucleation.

It is well known that ultrasonic melt treatment (UST) effectively enhances heterogeneous nucleation, leading to grain refinement^10–12^. Ultrasound-induced acoustic cavitation in a melt can generate a localized hot spot with temperatures of approximately 5000 °C and pressures of about 500 atmospheres when the cavitation bubbles collapse^13,14^. The increase in local temperature and pressure also generates a shock wave and acoustic streaming, mixing the melt^14^. In case of inoculation with UST, the ultrasonically induced shock wave and acoustic streaming can de-agglomerate and disperse inoculant particles, increasing the number of nucleation events^10,11^. Han *et al*.^15^ observed that UST uniformly dispersed TiB_2_ particles in a melt of Al-5Ti-1B; they suggested that grain refinement by UST is due to the de-agglomeration effect. Sreekumar *et al*.^11^ reported for an A357 alloy + Al-1.5B-7.6Al_2_O_3_ master alloy that grain refinement by UST was due to improved wettability and dispersion of Al_2_O_3_ oxide particles formed by *in-situ* reaction. Even in the absence of inoculants, the increase in local pressure and temperature by UST are also suggested to improve the wettability of non-wetting oxide particles, which are naturally present in an Al melt, thereby enhancing the heterogeneous nucleation of α-Al grains^14,16^.

However, controversial results on grain refinement by UST have also been reported. That is, UST does not always lead to grain refinement for aluminum alloys. Zhang *et al*.^4^ reported that the UST refined grain size of AA7075 and AA2024 alloys at cooling rate of 2.0 K/s if nucleant particles are Al_3_(Zr, Ti), while UST increased the grain size if TiB_2_ particles are provided as nucleation substrates. Puga *et al*.^17^ reported that the grain refinement efficiency decreases with increasing melt temperature for UST applied to Al-9Si-3Cu alloy. In addition, in our recent study^5^, UST at about 100 °C above the liquidus temperatures of hypoeutectic Al-Si alloys with Cu and Mg additions was found to increase the grain size, although a large number of MgAl_2_O_4_ particles were observed to be well wetted and finely dispersed. The above research works suggest that the increase in the grain size is possibly due to de-agglomeration of particle by UST, inducing decrease in size of inoculant particles for α-Al, which increases the required undercooling for nucleation.

However, up to now, a quantitative approach accounting for the relationship between the size and distribution of nucleant particles and the grain size has not been yet attempted. In addition, the critical influence of melt temperature on ultrasonic grain refinement is not clearly understood. Therefore, in this study, we aim to verify more quantitatively the relationship between the number of potent particles and the critical size with melt temperature for UST by measuring the particles size and their distribution, and to suggest a condition in which grain refinement by UST can be achieved.

Meanwhile, Mg is often added to Al alloys to improve the mechanical properties while its addition always causes the formation of MgO and MgAl_2_O_4_ oxides in the alloy melt^5,18^. Fan *et al*.^2,19^ developed a practical melt process, namely, intensive melt shearing and attempted to utilize such oxide particles as potent nucleants. Having a very small lattice misfit of 1.4% with α-Al phase, MgAl_2_O_4_ particles with their significantly increased number density had strong dispersive power, thus contributing to the grain refinement of Al-Mg alloys^2,19,20^. Harini *et al*.^20^ reported that UST resulted in a 7–8 fold reduction in grain size of an Al-1Mg-0.1SiO_2_ alloy. They suggested that ultrasonic cavitation accelerated the SiO_2_ reduction reaction, increasing the number density of MgAl_2_O_4_ particles and their wettability, while acoustic streaming dispersed the active nuclei throughout the melt^20^. As mentioned above, in our previous study^5^, although a relatively a large number of MgAl_2_O_4_ particles formed in the melt due to the relatively higher Mg content (1wt.%) in the Al-7Si-2Cu-1Mg alloy, grain refinement did not occur, so it is necessary to clarify the reason and it is thought to be suitable for the purpose of this study. Thus, the Al-7Si-2Cu-1Mg alloy was used in this study.

## Results

### Observation of MgAl_2_O_4_ particles

**Figure 1:**
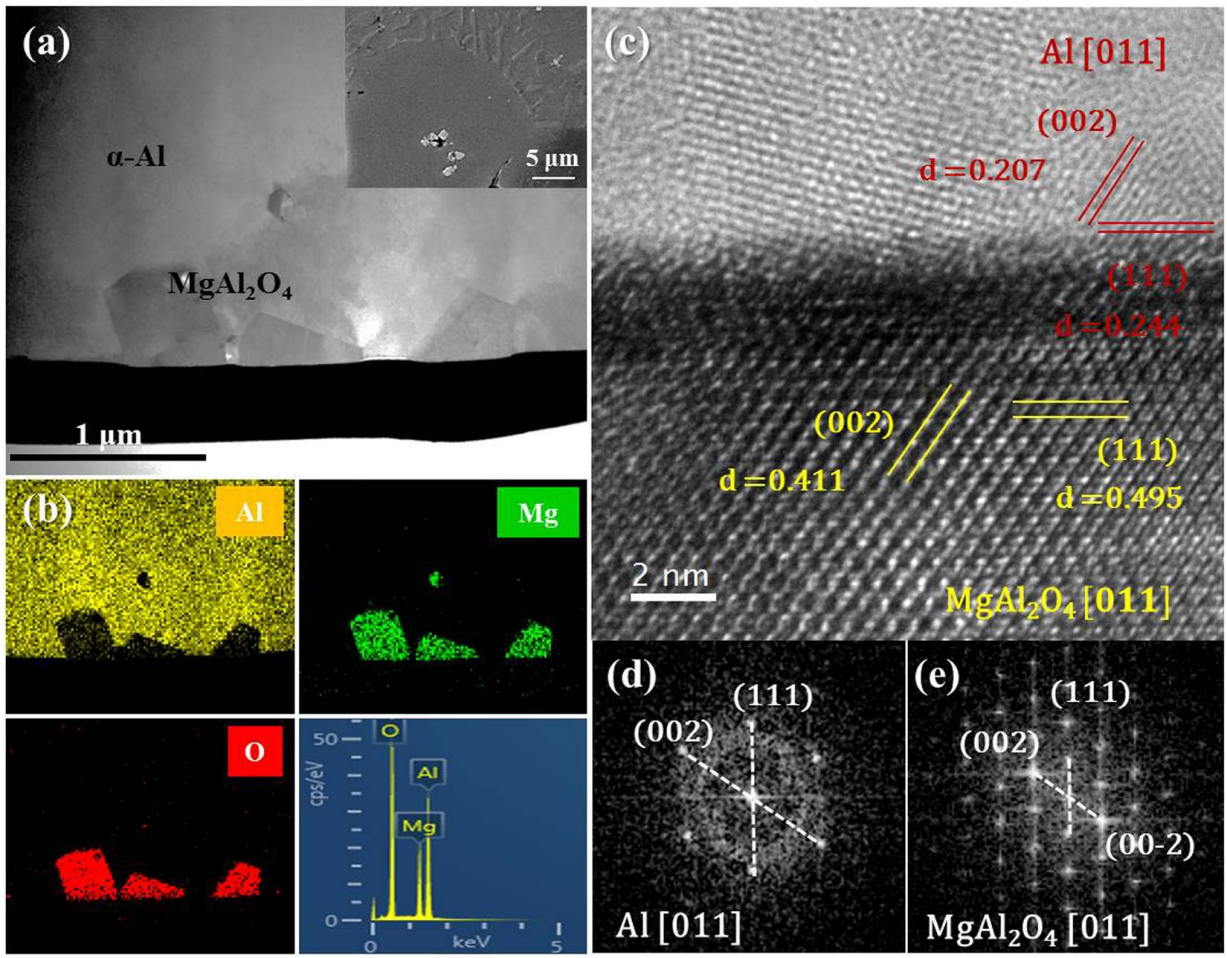
(**a**) Bright field TEM micrograph and (**b**) corresponding EDS maps and spectrum exhibiting faceted MgAl_2_O_4_ particles embedded in α-Al grain of Al-7Si-2Cu-1Mg alloy water-quenched with UST at 620 °C. SEM showing particles is inset of (**a**). (**c**) High resolution TEM image showing MgAl_2_O_4_/α-Al interface and SAD patterns of (**d**) α-Al and (**e**) MgAl_2_O_4_, both having [011] zone axis.

In order to discuss the effect of number and size of the particles on grain refinement by UST for the alloy, potency of MgAl_2_O_4_ for Al nucleation should first be confirmed. Figure 1 shows a TEM image of MgAl_2_O_4_ particles embedded in Al matrix of USTed alloy. MgAl_2_O_4_ particles with a faceted morphology were found to be present within α-Al grains (see inset of Fig. 1(a)); a thin foil TEM sample containing the area of interest was prepared by a JEOL JIB-4601F focused ion beam (FIB). TEM imaging (Fig. 1(a)) along with EDS analysis (Fig. 1(b)) confirmed that the particles embedded in the α-Al grain were MgAl_2_O_4_. A high resolution TEM image (Fig. 1(c)) and selected area diffraction (SAD) patterns (Fig. 1(d,e)) obtained by Fast Fourier Transformation (FFT) analysis clearly indicate that both the α-Al and MgAl_2_O_4_ are oriented along the [011] zone axis direction and that MgAl_2_O_4_ (111) and α-Al (111) are parallel to each other. (111) d spacing for both MgAl_2_O_4_ and Al was measured from the FFT images of Fig. 1(d,e), respectively and the results are indicated in Fig. 1(c). The identified orientation relationship is established as (111)[011] MgAl_2_O_4_//(111)[011] α-Al, as reported in the literature^2,19^. The calculated inter-planar misfit between the (111) planes of α-Al and MgAl_2_O_4_ is 1.4%, which is much less than the suggested 10% maximum for potent nuclei^21^. Therefore, it is confirmed that MgAl_2_O_4_ is a potent nucleation site for the α-Al of the alloy.

### Grain size

**Figure 2:**
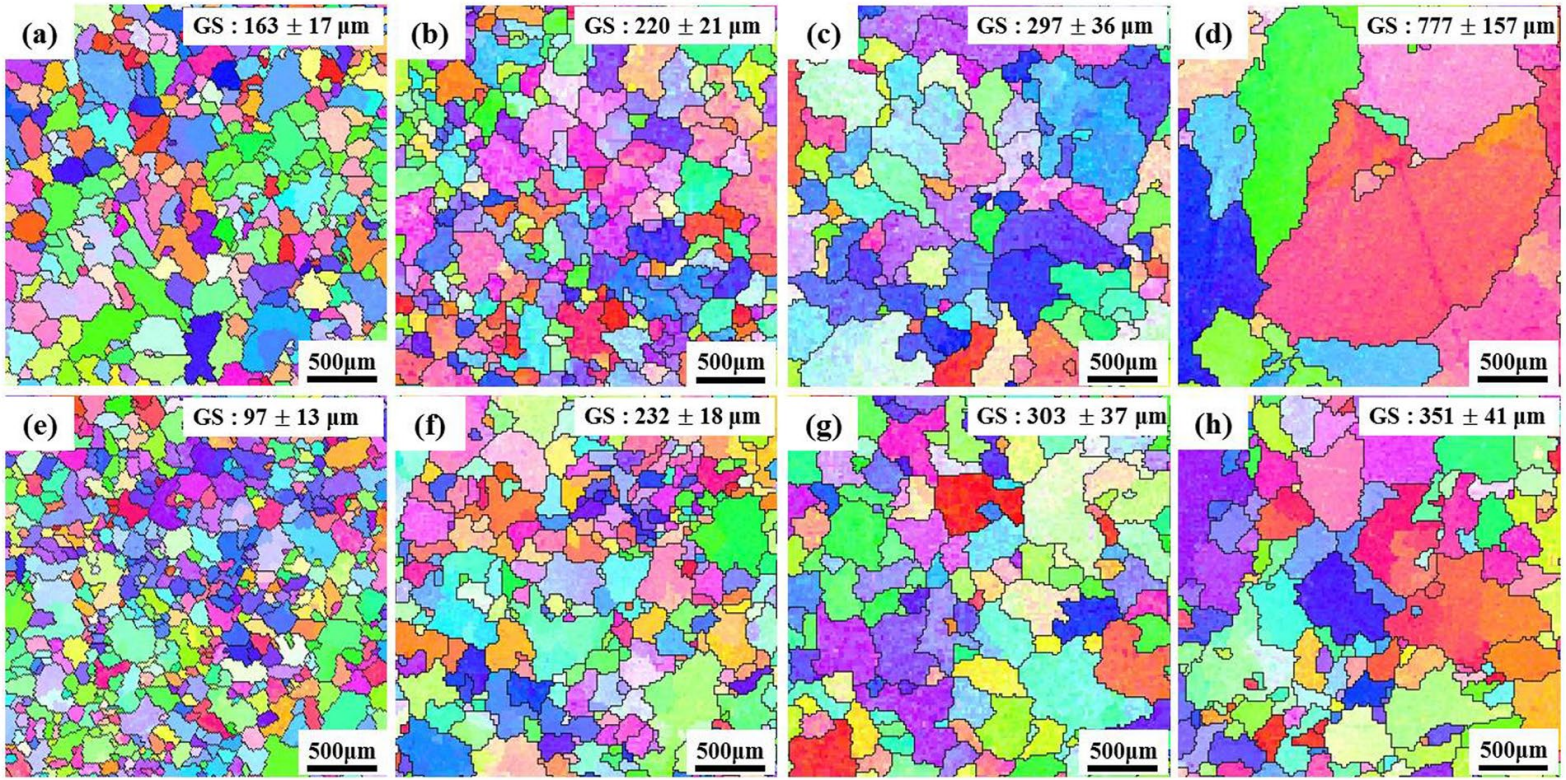
EBSD maps exhibiting grains of Al-7Si-2Cu-1Mg alloys (**a**–**d**) without UST and (**e**–**h**) with UST solidified at different pouring temperatures of (**a**,**e**) 620 °C, (**b**,**f**) 650 °C, (**c**,**g**) 700 °C and (**d**,**h**) 785 °C.

**Figure 3:**
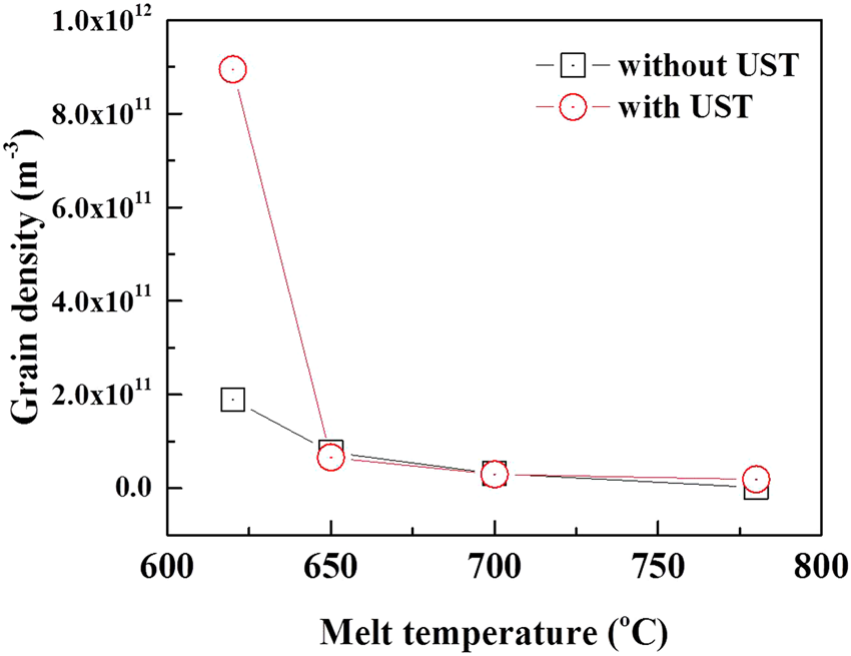
Calculated number density of grains nucleated as a function of melt temperature.

Figure 2 shows EBSD maps exhibiting grains of the Al-7Si-2Cu-1Mg alloys poured at melt temperatures of 620, 650, 700 and 785 °C. In alloys both with and without UST, the grain size (GS) increases with increasing temperature, but its dependence on the melt temperature is less pronounced for the alloy with UST than for alloy without UST. It is also interesting to note that UST at 620 °C most effectively reduced the grain size to 97 μm and that ultrasonic grain refinement became insignificant at melt temperatures exceeding 650 °C. The calculated number density of grains nucleated for the alloys with and without UST was illustrated in in Fig. 3. In this study, the volumetric grain density which can be converted from areal grain density was employed in order to account for the nucleation event occurring at each different melt temperature. If the spatial grain configuration is assumed to satisfy a Voronoi model, the volumetric grain density can be calculated using following equation^9^.

2 $${N}_{v}=0.568\,{(N)}^{3/2}$$

where *N*_*v*_ is the volumetric grain density, *N* is the areal grain density. *N* was a number of grains nucleated per 1 m2 (*N* = 1/grain size) and the grain shape was assumed to be a circle. The constant, 0.568, is a coefficient to convert to spatial density. Figure 3 indicates that a significant increase in the grain density can only be achieved when UST is conducted at 620 °C, just above the liquidus temperature (607 °C). For melt temperatures of 650–785 °C, however, nearly the same number of grains formed in alloys both with and without UST.

### Thermal analysis

**Figure 4:**
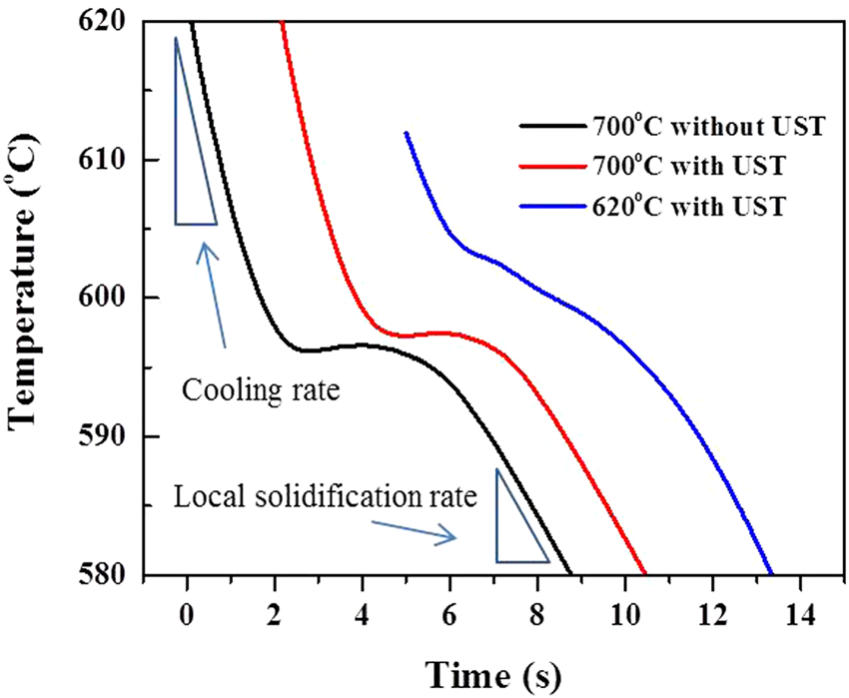
Cooling curves of the alloys without and with UST.

Figure 4 shows cooling curves of the alloys without and with UST. These cooling curves show that the melt temperature has little effect on the cooling rate or local solidification rate. Unfortunately, nucleation temperature was difficult to define by differential method due to fast cooling by permanent Cu-mold casting. When the melts were poured at 700 °C, maximum undercooling defined as Δ*T*_*m*_ = *T*_*g*_ *− T*_*min*._ of the alloys without and with UST, wer 0.4 and 0.3 °C respectively, which are a similar values. When UST is applied to the melt at 620 °C, it is confirmed that no recalescence was observed and α-Al nucleation temperature range was elevated even though *T*_*min*._ was not clearly defined. The increase in the nucleation temperature range suggests that UST at 620 °C more effectively promotes heterogeneous nucleation, which is in good agreement with the results of the observation of microstructures for the grain size. According to the free growth theory, nucleation first occurs on the largest nucleant particle and finally stops when recalescence starts at *T*_*min*._ (the temperature at which the smallest activated particle nucleates). Therefore, at 620 °C, the elevated nucleation temperature range is possibly related to size and number of MgAl_2_O_4_ particles.

### Size and its distribution of MgAl_2_O_4_

**Figure 5:**
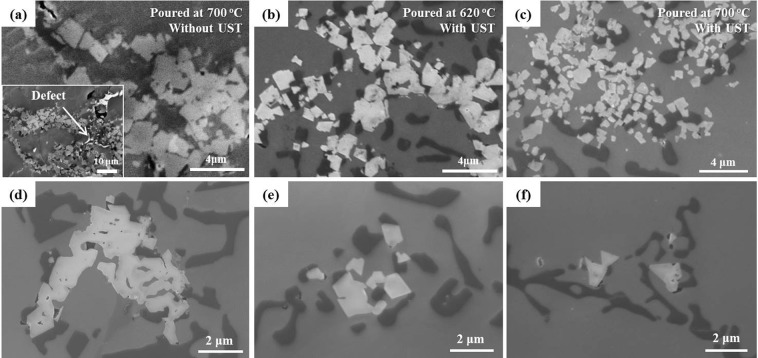
SEM images of MgAl_2_O_4_ particles observed in Al-7Si-2Cu-1Mg alloys (**a**) without UST at 700 °C, (**b**) with UST at 620 °C and (**c**) with UST at 700 °C. (**d**) to (**f**) The particles of various shapes and sizes present in the alloy with UST at 620 °C (**d**) agglomeration of MgAl_2_O_4_ particle, (**e**) and (**f**) dispersed individual MgAl_2_O_4_ particles.

**Figure 6:**
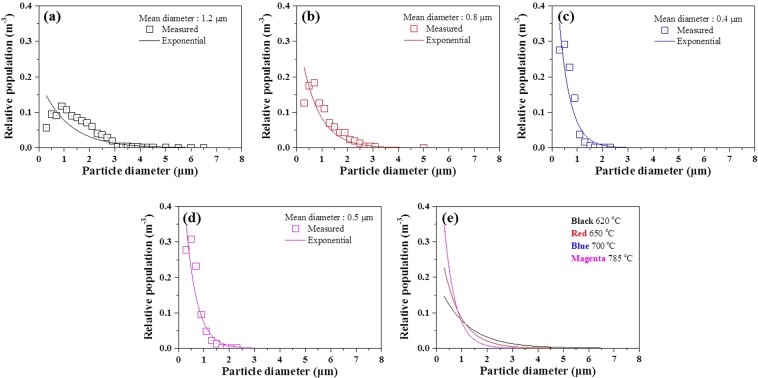
Quantitative analysis of MgAl_2_O_4_ particle sizes measured for alloys with UST at (**a**) 620, (**b**) 650, (**c**) 700 and (**d**) 785 °C. (**e**) Exponential distribution of particle sizes of all alloys with UST.

Figure 5 shows SEM images of MgAl_2_O_4_ particles present in the alloys without and with UST. Without UST, MgAl_2_O_4_ particles are mostly observed in the form of relatively coarse agglomerations of the particles attached to oxide film (Fig. 5(a)). In addition, defects along the oxide films are observed, which implies that MgAl_2_O_4_ particles are not well wetted. On the other hand, with UST, such oxide films are broken up and MgAl_2_O_4_ particles with faceted morphologies were finely dispersed into individual particles, as can be observed in Fig. 5(b–f). Without UST, MgAl_2_O_4_ particles present in the alloy are basically in a form of coarse agglomerations (Fig. 5(a)) in which individual particles are attached to each other. However, as UST breaks them up, the agglomeration size was decreased. Accordingly, the MgAl_2_O_4_ particles can be present in a form of relatively small size agglomeration and/or individual particles as shown in Fig. 5(d–f). The de-agglomeration process is assumed to be following procedure: oxide film (Fig. 5(a)) → a relatively small size of agglomerated form (Fig. 5(d)) → individual particles (Fig. 5(e,f)). This is thought to be enhanced with an increase in the melt temperature for UST, which accordingly decreased the number of the agglomeration. A detailed quantitative analysis on the MgAl_2_O_4_ particles is shown in Fig. 6. Unfortunately, without UST, the quantitative analysis was not performed due to difficulty in the collection of individual particle information because the MgAl_2_O_4_ particles were not well wetted and mainly exist in oxide film form.

**Figure 7:**
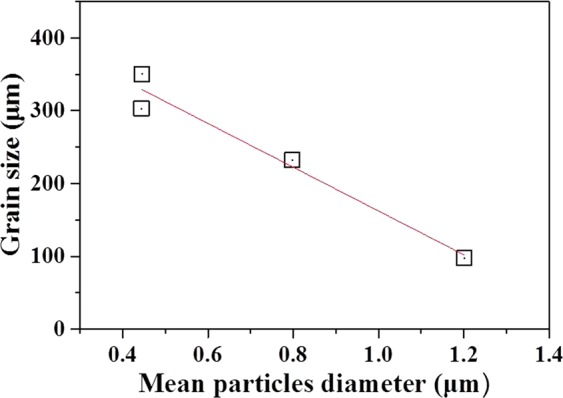
Measured grain size as function of mean MgAl_2_O_4_ particle size.

Quantitative analysis of inoculant particle size has already been performed for various inoculant particles^6,7,9,11^. In the case of the addition of Al-Ti-B master alloys, TiB_2_ particles are thought of as nucleation sites for α-Al grains. It should be noted that smaller particles size below 200 nm make it difficult to establish the population distribution since scratches and other surface detects may be wrongly interpreted by image analysis^6,7,9^. Hence, various probability density distribution models such as exponential distribution^6^, log-normal distribution^7^, Weibull distribution^9^, etc., are employed. Among them, the exponential distribution model is most well matched with the results measured in this study. Hence, the exponential distribution model was employed in this work and the sizes of particles were fitted using the following probability density function

3 $$f(d)=\lambda \cdot \exp (-\lambda d)$$

where *d* is diameter of the particles, *d*_*a*_ is mean diameter of the particles and *λ* is a distribution parameter given as 1/*d*_*a*_. As shown in Fig. 6, the exponential size distribution fits well with the measured size distribution. The size distribution shows that increasing the melt temperature for UST from 620 to 785 °C significantly reduces the mean particles diameter from approximately 1.2 to 0.4 μm. Figure 7 shows the relationship between the grain size and the mean particles diameter. Though the decrease in the particle size is expected to lead to an increase in the number of particles when total the particles volume is constant, the grain size instead increases with decreasing the particle size. According to the free growth theory, the nucleant particle size strongly affects the number of the potent particles since undercooling required for nucleation is inversely proportional to the particle size (Eq. 1).

The exponential distribution of MgAl_2_O_4_ particle size with mean particle size can provide information on number of the particles. Equation 3 can be modified as follows

4 $${N}_{tot}\cdot f(d)=\frac{{N}_{tot}}{{d}_{a}}\cdot \exp (-\,\frac{d}{{d}_{a}})$$

where *N*_*tot*_ is total number of MgAl_2_O_4_ particles present in the alloy with a weight of 1.5 kg. Only particles having a size greater than a critical diameter, d*, can be activated for nucleation, so that the number of the activated particles, *N*_*n*_, can be obtained by integrating Eq. 4, as follows

5 $${N}_{n}={\int }_{d\ast }^{\infty }{N}_{tot}\cdot f(d)dd={N}_{tot}\cdot \exp (-\frac{{d}^{\ast }}{{d}_{a}})$$

Fraś *et al*.^9^ fitted TiB_2_ particles size distribution (that is, *d*_*a*_ = constant) for different degrees of undercooling. However, in this work, the shape of particle size distribution varies according to the melt temperature (that is, *d*_*a*_and *N*_*tot*_ are different for each melt temperature), so Eq. 5 should be modified. Haginoya *et al*.^22^ reported that the mass gain by oxidation for formation of MgAl_2_O_4_ was not significant when Al melt containing less than 2 wt.% of Mg is oxidized for 3 hrs,; in addition, the mass gain hardly changed until the melt temperature reached 800 °C. In this work, because all melts were held at the same temperature, 800 °C, for the same time, 2 hrs, the total volume of MgAl_2_O_4_, *V*_*tot*_, in each melt temperature can be assumed to be constant. Because individual MgAl_2_O_4_ has an equiaxed polygonal shape as shown in Fig. 5, the mean volume of the particles can be calculated as *d*_*a*_^3^, assuming the shape of the particles are, for simplicity, cubic. Hence, the total volume of the particles can be expressed as *N*_*tot*_ = *V*_*tot*_/*d*_*a*_^3^. The nucleation parameters estimated based on the experimental results can be fitted to the logarithmic form of Eq. 5 yielding a linear relationship between ln(*N*_*n*_*∙d*_*a*_^3^) and 1/*d*_*a*_

6 $$\mathrm{ln}({N}_{n}\cdot {d}_{a}^{3})=\,\mathrm{ln}\,{V}_{tot}-\frac{{d}^{\ast }}{{d}_{a}}$$

**Figure 8:**
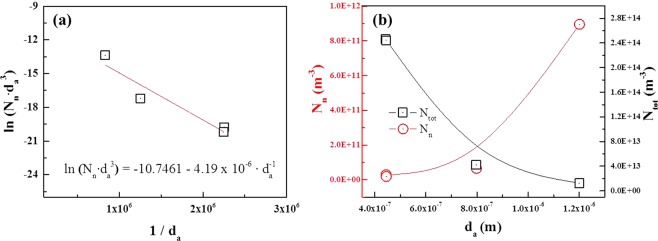
Relationship between mean diameter of MgAl_2_O_4_ particles and number of activated particle; (**a**) inverse of mean diameter of particles, 1/d_a_, vs. total volume of activated particles, ln(N_n_∙d_a_^3^). (**b**) Mean diameter of particles, d_a_, vs. total number of activated particles, N_n_.

The experimental data on *d*_*a*_ and *N*_*n*_*∙d*_*a*_^3^ for melt temperatures of 620, 650, 700 and 785 °C are plotted as ln(*N*_*n*_*∙d*_*a*_^3^) and 1/*d*_*a*_. The *V*_*tot*_ and d^*^ are determined from the straight line plots as shown in Fig. 8(a). All the nucleation parameters obtained by this fitting are shown in Table 1. It is difficult to quantitatively measure the total number of oxide particles present in the melt but, the total number of the particles can be approximately estimated using this plot. This plot shows that values of ln*V*_*tot*_ and d* are −10.7461 and 4.19 × 10^−6^   (4.19 μm), respectively. Values of *V*_*tot*_ and *N*_*tot*_ are calculated as *e*^−10.7461^ = 2.15 × 10^−5^ m^3^ for all the melt temperature and 1.24 × 10^13^, 4.24 × 10^13^, 2.46 × 10^14^ and 2.44 × 10^14^ m^−3^ for each melt temperature of 620, 650, 700 and 785 °C respectively. The fitting results show that only particles with sizes greater than 4.19 μm can be active for nucleation. That is, under UST, taking into account the measured size distribution, the largest number of MgAl_2_O_4_ particles can be activated at 620 °C (Fig. 6). This is in good agreement with the significant increase in grain density at 620 °C (Fig. 3). Figure 8(b) shows the inverse relationship between total number of the particles, *N*_*tot*_, and activated number of the particles, *N*_*n*_. In other words, as the mean diameter of particle size decreases, the total number of particles increases significantly, but the number of activated particles for Al nucleation significantly decreases.

**Table 1:** Nucleation parameters obtained by fitting of Eq. 6.

Temp. (°C)	*Grain density (N_n_), m^3^	Mean size of MgAl_2_O_4_		λ (1/d_a_), m^−3^	V_n_, m^3^	ln(N_n_∙d_a_^3^) (=lnV_n_)	d*, m	Intercept (ln V_tot_)	V_tot_, m^3^	N_tot_, m^−3^
		Diameter (d_a_), m	Volume (d_a_^3^), m^3^							
620	8.95 × 10^11^	1.20 × 10^−6^	1.73 × 10^−18^	8.33 × 10^5^	1.60 × 10^−6^	−13.38	4.19 × 10^−6^	−10.75	2.15 × 10^−5^	1.24 × 10^13^
650	6.54 × 10^10^	0.79 × 10^−6^	5.08 × 10^−19^	1.25 × 10^6^	6.19 × 10^−8^	−17.22				4.24 × 10^13^
700	2.94 × 10^10^	0.44 × 10^−6^	8.75 × 10^−20^	1.08 × 10^7^	2.57 × 10^−9^	−19.78				2.46 × 10^14^
785	1.89 × 10^10^	0.45 × 10^−6^	8.81 × 10^−20^	1.08 × 10^7^	1.67 × 10^−9^	−20.21				2.44 × 10^14^

^*^Grain densities were calculated based on grain size (Figs. 2 and 3).

## Discussion

From Fig. 1, it was confirmed that MgAl_2_O_4_ can nucleate α-Al. However, for the particles to contribute to grain refinement, sufficient wetting and number of the particles are both required. In general, oxide particles are considered as inclusions that cause casting defects due to their poor wettability^23^. As shown in the inset of Fig. 5(a), without UST, the oxide film was always found to exist near casting defects. This indicates that MgAl_2_O_4_ particles are naturally poorly wetted. On the other hand, with UST, no defects near the particles were observed and the particles show facetted morphology, which implies that the particles are well wetted by UST. It is believed that an increase in local pressure and temperature by cavitation bubble collapse can clean the particle surface and reduce the surface tension of Al, improving the wettability^14,16^. It is therefore thought that UST improves the wettability of the particles, increasing the number of MgAl_2_O_4_ particles.

UST can also de-agglomerate the MgAl_2_O_4_ particles, increasing the total number of the particles. Li *et al*.^2^ suggested that if an external force is provided for a melt to overcome the capillary pressure, not only can the wettability of MgAl_2_O_4_ particles be improved but also their de-agglomeration can be achieved. The shock wave and acoustic streaming induced by UST possibly provide the external force for the de-agglomeration. For this reason, MgAl_2_O_4_ particles are converted from the film form (Fig. 5(a)) to individual particle form (Fig. 5(e,f) by UST. According to Li *et al*.^2^, individual MgAl_2_O_4_ particles in Al-(0.7 to 5 wt.%) Mg alloys exhibits a sub-micron size (0.2–0.5 μm) and oxide films also consist of sub-micron sized individual particles. The mean particle size measured in this study is in a range of 1.2 to 0.4 μm, which is slightly above the sub-micron size. It is therefore considered that UST is unlikely to break individual particles into fragments and thus the decrease in the particle size is eventually attributed only to de-agglomeration.

More importantly, the de-agglomeration effect is also affected by melt temperature for UST as shown in Fig. 6. The mechanism of ultrasonic de-agglomeration has been suggested to involve the penetration of liquid metal into the agglomerates^2,24–26^, followed by implosion of cavitation bubbles, dispersing the agglomerates more uniformly in the melt^27^. This can be described by the ultrasonic capillary effect, which works in the melt only if the capillary pressure is overcome^27,28^.

7 $${P}_{c}=\frac{2{\gamma }_{LV}cos\theta }{r}$$

where *γ*_*LV*_ is the surface tension of the liquid alloy, *θ* is the contact angle, and *r* is the equivalent distance between oxide particles within agglomerations. Knowing that the surface tension of the melt is inversely proportional to the temperature, the capillary pressure can be reduced by increasing the melt temperature. Therefore, it is reasonably expected that UST at a higher melt temperature can separate MgAl_2_O_4_ agglomerates into individual particles more effectively due to the relatively lower capillary pressure. Thus, the grain size is possibly related to the particle size.

The relationship between grain size and the particle size was derived employing analytical model. Analytical models are known to make quantitatively accurate predictions for grain size in inoculated Al casting alloys^6,7^ while determining the important parameters affecting grain refinement such as critical diameter and numbers of nucleants particle activated for α-Al nucleation. Greer *et al*.^6^ and Quested *et al*.^7^ suggested an exponential distribution model and a log-normal distribution model, respectively to account for the relationship between TiB_2_ particle size and grain size with experimental measurement of particle size distribution. They determined a particular particle diameter at which the grain refinement efficiency is optimized. More recently, Fraś *et al*.^9^ using a Weibull distribution of particle size measured quantitatively, estimated critical diameter of TiB_2_ nucleant particles to trigger α-Al nucleation and the density of grain nuclei. Having considered the free growth model^6^, the Weibull distribution was modified in the form of Eq. 5, in which *d*_*a*_ is replaced with $\frac{4\gamma }{\Delta {S}_{v}\Delta {T}_{m}}$ to plot against measured undercooling, Δ*T*_*m*_, because d_a_ in their study was assumed to be constant.

**Figure 9:**
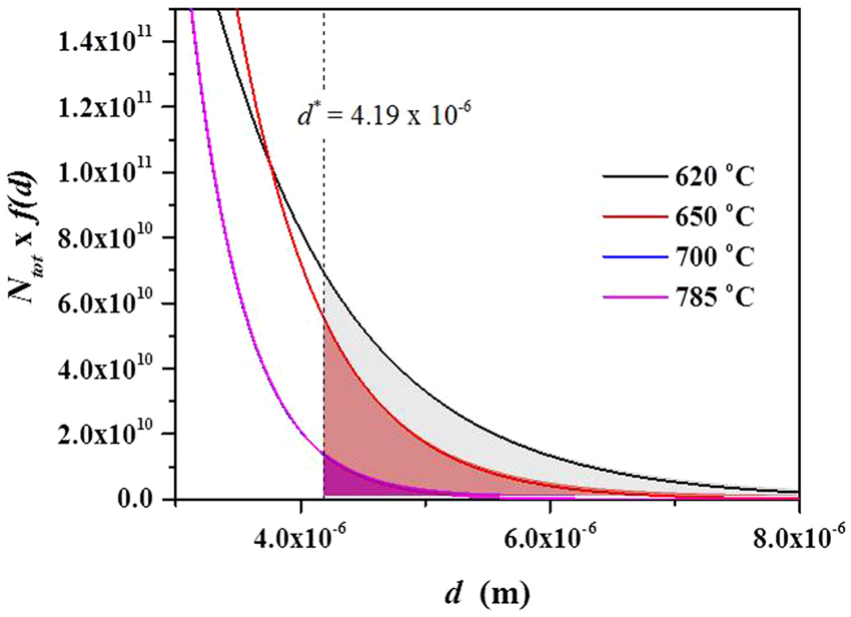
Distribution function showing the total number of particle sizes estimated by line fitting. Shaded areas under each curve indicate number of activated particles, *N*_*n*_, for α-Al nucleation.

Similar to a case of inoculation, the relationship between inoculant particle size and heterogeneous nucleation can also be applicable to the alloys with UST. In our study, however, it should be noted that we have employed an exponential distribution model in the form of Eq. 5 particularly considering the variation of *d*_*a*_ with UST temperature. The exponential probability density function (Eq. 3 and Fig. 6) indicates the relative number density of MgAl_2_O_4_ particles. Hence, using the estimated total number of the particles, *N*_*tot*_, the exponential distributions for different melt temperatures can be re-plotted as a function of *d* as shown in Fig. 9 (*N*_*tot*_ × *f*(*d*). From the *d* vs *N*_*tot*_ × *f*(*d*) curves of Fig. 9, the absolute number of activated particles for nucleation was clearly determined according to the areas and was found to decrease as the melt temperature increases. Here, it is worth noting that the estimated numbers of activated particles, N_n_, as determined by calculation, are in good agreement with the number obtained by measurement. The estimated numbers of activated particles are summarized in Table 2; the values of line-fitting were calculated by Eq. 5 using the shaded area in Fig. 9, and the grain density were obtained by measurement of grain size (Figs. 2 and 3). Therefore, this suggests that a linear plot against 1/*d*_*a*_ based on the size distribution is reasonable tool to explain grain refinement by UST.

**Table 2:** Number of activated particle estimated from grain density (Fig. 4) and using line fitting method (Fig. 10).

Temp. (°C)	Mean particle diameter, d_a_ (m)	Number of activated particle, N_n_ (m^−3^)	
		Grain density	Line fitting
620	1.20 × 10^−6^	8.95 × 10^11^	7.47 × 10^11^
650	0.79 × 10^−6^	6.54 × 10^10^	3.92 × 10^11^
700	0.44 × 10^−6^	2.94 × 10^10^	6.22 × 10^10^
785	0.45 × 10^−6^	1.89 × 10^10^	6.30 × 10^10^

Use of UST in molten metal has attracted considerable research attention as it has a significant effect on the structural refinement, particularly the grain refinement^4,10^. Up to now, several mechanisms for grain refinement by UST have been suggested and the following mechanisms are generally accepted. The first mechanism is known to be due to cavitation bubble expansion: the gas inside the bubbles rapidly expands during cavitation, thereby inducing undercooling at the bubble surface^29^. The second mechanism is explained by the Clapeyron equation,: pressure pulse generated by bubble collapse increases melting point of melt and consequently increases undercooling^30^. The third mechanism is known as cavitation-enhanced wetting of oxide particles^3,16^. The intensive heat and pressure generated by bubble collapse are suggested to increase the surface reaction^3^ and thus enhance the wettability of non-wetting oxide particles that are always present in the Al alloy melt^16^.

Among the above mechanisms, it is thought that the mechanism of cavitation-enhanced wetting of oxide particles can be used to clearly interpret the results of this study. Previous studies^6,7,9^ have clearly shown the relationship between inoculant particle size and heterogeneous nucleation when the melt is inoculated (in the absence of UST). Similar to the case of inoculation, that relationship can also be applicable to an alloy with UST. As explained by the experimental measurements (see Figs. 5 and 6), UST changes nucleant particle (MgAl_2_O_4_ oxide) size and its distribution, affecting heterogeneous nucleation. Knowing that ultrasonic de-agglomeration is further enhanced by increases in t melt temperature, at first glance, it would simply be expected that an increase in the number density of MgAl_2_O_4_ particles with size reduction at higher temperature could result in grain refinement. However, it is experimentally observed that optimized grain refinement can only be achieved through a certain condition in which UST provides the largest contribution to heterogeneous nucleation. In other words, along with the wettability, a certain diameter of MgAl_2_O_4_ particles should be reached for free growth of α-Al grains. From the fitting results (Fig. 8 and Table 1), the critical diameter was calculated and found to be 4.19 μm at a given undercooling. This means that only particles with sizes greater than the critical diameter, d*, 4.19 μm can be activated for α-Al nucleation. As indicated in Fig. 9, with increase of the temperature for UST, the number of the particles exceeding d* decreased due to the de-agglomeration effect. Thus, grain refinement by MgAl_2_O_4_ particles is only achieved when UST is applied at 620 °C as can be seen in Fig. 2.

The quantitative approach suggested in the present work may also account for other controversial results^4,5,17^ regarding grain refinement by UST. Zhang *et al*.^4^ suggested that the de-agglomeration effect of UST could diminish the grain refinement. In their study, UST refined the grain size of AA7075 and AA2024 alloys at a cooling rate of 2.0 K/s if potential inoculant particles were Al_3_(Zr, Ti), while UST increased the grain size of the alloys inoculated with TiB_2_. This has been explained as attributable to the difference in mean particle size between Al_3_Ti and TiB_2_ nucleants, which were refined by UST to 3–8 μm and 1 μm, respectively^4^. It was also suggested that Al_3_Ti phases were still large enough to nucleate α-Al grain with undercooling of 0.2 K^4^. However, compared to the case of coarse Al_3_Ti phases, TiB_2_ particles finely dispersed with size of approximately 1 μm would significantly increase required undercooling for nucleation and hence become ineffective for grain refinement^4^. This is consistent with our results, which showed that most finely dispersed MgAl_2_O_4_ particles with UST were not activated for α-Al nucleation, while only a very few MgAl_2_O_4_ particles exceeding the critical diameter became activated. Besides the nucleant particle size, the melt temperature is an important factor affecting the ultrasonic grain refinement performance. Puga *et al*.^17^ reported that grain refinement was only achieved at melt temperatures slightly above the liquidus temperature of Al-9Si-3Cu alloy with UST, while grain refinement at different melt temperatures has been explained by the different thermal stability of acoustically induced nuclei^17^; however, these issues are hardly discussed from the viewpoint of nucleant particle size.

Even in the absence of inoculation particles, oxide particles are present in an Al melt and are often agglomerated in nature. As evidenced in Figs. 5 and 6, UST involving acoustic streaming can disperse oxide aggregates into individual particles, of which the mean size decreases as the melt temperature for UST increases. However, increase in the number density of nucleating particles by ultrasonic de-agglomeration does not always induce grain refinement. High potency nucleant particles with relevant size and distribution for triggering α-Al nucleation are indeed of importance and are necessarily required for the realization of grain refinement by UST.

## Method

**Figure 10:**
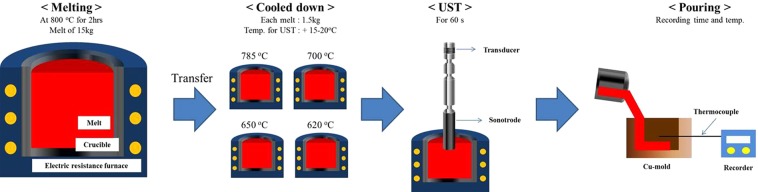
Schematic diagram showing experimental procedure.

A schematic diagram of sample preparation is shown in Fig. 10. Al-7Si-2Cu-1Mg alloy was prepared by melting a commercial 15 kg A356 alloy, followed by the addition of commercial purity Cu (99.9%) and Mg (99.9%) at 800 °C in an electric resistance furnace (See the chemical composition presented in Table 3). This melt was held at 800 °C for 2 hrs in an electric resistance furnace to produce the same total volume of MgAl_2_O_4_. After stabilization, degassing treatment with Ar-gas bubbling filtration was carried out. Individual melts weighing 1.5 kg were transferred from the furnace into clay graphite crucibles placed in furnace at 620, 650, 700 and 785 °C and all the melts were isothermally held for 0.5 hrs respectively in the furnace. In parallel, melts for UST were held at 640, 670, 720 and 800 °C for 0.5 hrs, which led to an instant temperature drop of approximately 15–20 °C due to sonotrode immersion, followed by UST for 60 s using a SiAlON sonotrode. All melts were then poured into a Cu block mold preheated to 150 °C (cooling rate is approximately 5 K/s). For thermal analysis, K-type thermocouple was embedded in the center of mold through the mold cavity. The temperature and time were recorded using a computer-aided recorder (MV1000, YOKOGAWA).

**Table 3:** Chemical compositions of investigated alloys [wt.%].

Pouring temperature (°C)	UST	Si	Fe	Cu	Mn	Mg	Ti	Zr	Al
620	X	6.9	0.14	2.1	0.006	1.12	0.02	0.01	Bal.
	O	6.8	0.15	2.1	0.005	1.08	0.02	0.01	Bal.
650	X	6.8	0.14	2.2	0.006	1.11	0.02	0.01	Bal.
	O	6.7	0.14	2.2	0.004	1.08	0.02	0.01	Bal.
700	X	6.8	0.13	2.1	0.005	1.09	0.02	0.01	Bal.
	O	6.9	0.15	2.1	0.005	1.07	0.02	0.01	Bal.
785	X	7.1	0.16	2.1	0.005	1.05	0.02	0.01	Bal.
	O	6.8	0.16	2.0	0.005	1.06	0.02	0.01	Bal.

MgAl_2_O_4_ particles were observed using a ZEISS, Merlin Compact scanning electron microscope (SEM) and a JEOL-JEM 2100 F transmission electron microscope (TEM) equipped with an energy dispersive X-ray spectrometer(EDS). In particular, to analyse orientation relationship with Al matrix, MgAl_2_O_4_ particles were collected from water quenched sample USTed at 620 °C. The grain sizes of samples were determined by electron backscatter diffraction pattern (EBSD) in conjunction with a Tescan MIRA I LMH field emission gun SEM (FE-SEM). Image analysis using I-solution DT software was employed to measure the size of the MgAl_2_O_4_ particles.

## Conclusions

1. With UST, well wetted MgAl_2_O_4_ particles are embedded in Al matrix and have a certain orientation relationship with the Al matrix, indicating that UST can promote heterogeneous nucleation by improving wettability of MgAl_2_O_4_ particles.
2. When UST was applied at 620 °C, grain refinement was achieved by well wetted MgAl_2_O_4_ for Al-7Si-2Cu-1Mg alloys. On the other hand, UST at temperatures higher than 650 °C did not lead to grain refinement despite well wetted MgAl_2_O_4_ particles being observed. As the melt temperature for UST increased, the grain size increased and the grain refinement performance decreased.
3. The quantitative analysis indicated that the mean size of the MgAl_2_O_4_ particles decreased from 1.2 to 0.4 μm with increase of the melt temperature from 620 to 785 °C.
4. From exponential distribution fitting against mean size of MgAl_2_O_4_ particles, critical diameter for α-Al nucleation was estimated to be 4.19 μm. In addition, the exponential distribution fitting indicates that the reduction in size of MgAl_2_O_4_ particle from 1.2 to 0.4 μm increased the total number of the particles from 1.24 × 10^13^ to 2.44 × 10^14^ m^−3^, but rather decreased the number of activated particles from 7.47 × 10^11^ to 6.30 × 10^10^ m^−3^. This importantly suggests that ultrasonic grain refinement can be achieved when number of nucleant particles greater than critical diameter is sufficient at given undercooling, indicating that reduced grain refinement performance with increasing melt temperature is possibly related to reduced MgAl_2_O_4_ particle size.

## Data Availability

All data generated or analysed during this study are included in this published article. Additional requests related to this article can be accessed by contacting the corresponding author.
